# Efficient DNA-based data storage using shortmer combinatorial encoding

**DOI:** 10.1038/s41598-024-58386-z

**Published:** 2024-04-02

**Authors:** Inbal Preuss, Michael Rosenberg, Zohar Yakhini, Leon Anavy

**Affiliations:** 1 School of Computer Science, Reichman University, 4610101 Herzliya, Israel; 2 Institute of Nanotechnology and Advanced Materials, The Mina and Everard Goodman Faculty of Life Sciences, Bar-Ilan University, 5290002 Ramat Gan, Israel; 3 Faculty of Computer Science, Technion, 3200003 Haifa, Israel

**Keywords:** Computational biology and bioinformatics, Information technology, Computational science, Computer science, DNA computing, DNA and RNA, Computational methods

## Abstract

Data storage in DNA has recently emerged as a promising archival solution, offering space-efficient and long-lasting digital storage solutions. Recent studies suggest leveraging the inherent redundancy of synthesis and sequencing technologies by using composite DNA alphabets. A major challenge of this approach involves the noisy inference process, obstructing large composite alphabets. This paper introduces a novel approach for DNA-based data storage, offering, in some implementations, a 6.5-fold increase in logical density over standard DNA-based storage systems, with near-zero reconstruction error. Combinatorial DNA encoding uses a set of clearly distinguishable DNA shortmers to construct large combinatorial alphabets, where each letter consists of a subset of shortmers. We formally define various combinatorial encoding schemes and investigate their theoretical properties. These include information density and reconstruction probabilities, as well as required synthesis and sequencing multiplicities. We then propose an end-to-end design for a combinatorial DNA-based data storage system, including encoding schemes, two-dimensional (2D) error correction codes, and reconstruction algorithms, under different error regimes. We performed simulations and show, for example, that the use of 2D Reed-Solomon error correction has significantly improved reconstruction rates. We validated our approach by constructing two combinatorial sequences using Gibson assembly, imitating a 4-cycle combinatorial synthesis process. We confirmed the successful reconstruction, and established the robustness of our approach for different error types. Subsampling experiments supported the important role of sampling rate and its effect on the overall performance. Our work demonstrates the potential of combinatorial shortmer encoding for DNA-based data storage and describes some theoretical research questions and technical challenges. Combining combinatorial principles with error-correcting strategies, and investing in the development of DNA synthesis technologies that efficiently support combinatorial synthesis, can pave the way to efficient, error-resilient DNA-based storage solutions.

## Introduction

DNA is a promising media storage candidate for long-term data archiving, due to its high information density, long-term stability, and robustness. In recent years, several studies have demonstrated the use of synthetic DNA for storing digital information on a megabyte scale, exceeding the physical density of current magnetic tape-based systems by roughly six orders of magnitude^1,2^. Physical density is one of several quantitative metrics for evaluating the efficiency of DNA-based storage systems, measured by the data unit per gram of DNA. Another performance metric, which was introduced in^3^, is called logical density, refering to the amount of data encoded in each synthesis cycle. Since DNA synthesis is the main cost component in DNA-based storage systems, increasing the logical density is the main focus of this work.

Research efforts in the field of DNA-based storage systems have mainly focused on the application of various encoding schemes, while relying on standard DNA synthesis and sequencing technologies. These include the development of error-correcting codes for the unique information channel of DNA-based data storage^4–8^. Random access capabilities for reading specific information stored in DNA also require advanced coding schemes^9–11^. Yet, despite the enormous benefits potentially associated with capacity, robustness, and size, existing DNA-based storage technologies are characterized by inherent information redundancy. This is due to the nature of DNA synthesis and sequencing methodologies, which process multiple molecules that represent the same information bits in parallel. Recent studies suggest exploiting this redundancy to increase the logical density of the system, by extending the standard DNA alphabet using composite letters (also referred to as degenerate bases), and thereby encoding more than 2 bits per letter^12,13^. In this approach, a composite DNA letter uses all four DNA bases (A, C, G, and T), combined or mixed in a specified predetermined ratio $\sigma =({\sigma }_{A},{\sigma }_{C},{\sigma }_{G},{\sigma }_{T})$. A resolution parameter $k={\sigma }_{A}+{\sigma }_{C}+{\sigma }_{G}+{\sigma }_{T}$ is defined, for controlling the alphabet size. The full composite alphabet of resolution $k$, denoted ${\Phi }_{k}$, is the set of all composite letters, so that ${\Sigma }_{i\in (A,C,G,T\}}{\sigma }_{i}=k$. Writing a composite letter is done by using a mixture of the DNA bases, determined by the letter’s ratio in the DNA synthesis cycle. Current synthesis technologies produce multiple copies, and by using the predetermined base mixture each copy will contain a different base, thus preserving the ratio of the bases at the sequence-population level.

While the use of numerical ratios supports higher logical density in composite synthesis, it also introduces challenges related to the synthesis and inference of exact ratios. Combinatorial approaches, which also consist of mixtures, address these challenges in a different way. Studies by Roquet et al. (2021) and Yan et al. (2023) contribute significantly to advancing DNA-based data storage technology. To encode and store data, Roquet et al. focus on a novel combinatorial assembly method for DNA. Yan et al. extend the frontiers of this technology by enhancing the logical density of DNA storage, using enzymatically-ligated composite motifs^13,14^.

In this paper, we present a novel approach for encoding information in DNA, using combinatorial encoding and shortmer DNA synthesis, leading to an efficient sequence design and improved DNA synthesis and readout interpretation. The method described herein leverages the advantages of combinatorial encoding schemes while relying on existing DNA chemical synthesis methods with some modifications. Using shortmer DNA synthesis also minimizes the effect of synthesis and sequencing errors. We formally define shortmer-based combinatorial encoding schemes, explore different designs, and analyze their performance. We use computer-based simulations of an end-to-end DNA-based data storage system built on combinatorial shortmer encodings, and study its performance. To demonstrate the potential of our suggested approach and experimentally test its validity, we performed an assembly-based molecular implementation of the proposed combinatorial encoding scheme and analyzed the resulting data. Finally, we discuss the potential of combinatorial encoding schemes and the future work required to enable these schemes in large-scale DNA-based data storage systems and other DNA data applications.

## Results

### Design of shortmer combinatorial encoding for DNA storage

We suggest a novel method to extend the DNA alphabet while ensuring near-zero error rates.

Let $\Omega$ be a set of DNA k-mers that will serve as building blocks for our encoding scheme. Denote the elements in $\Omega$ as ${X}_{1},\dots ,{X}_{N}$. Elements in $\Omega$ are designed to be sufficiently different from each other, to minimize mix-up error probability. Formally, the set is designed to satisfy $d\left({X}_{i},{X}_{j}\right)\ge d;\forall i\ne j$, with the minimal Hamming distance $d$ serving as a tunable parameter.

Other design criteria can be applied to the shortmers in $\Omega$, taking into consideration the properties of DNA synthesis, manipulation, and sequencing. These may include minimal Levenshtein distance, GC context, and avoiding long homopolymers. Clearly, any such filtering process will result in reduced alphabet size and reduced logical density.

Note that $N=\left|\Omega \right|\le {4}^{k}$. The elements in $\Omega$ will be used as building blocks for combinatorial DNA synthesis in a method similar to the one used for composite DNA synthesis^3^. Examples of k-mer sets $\Omega$ are presented in Supplementary Sect. 8.3.

**Figure 1:**
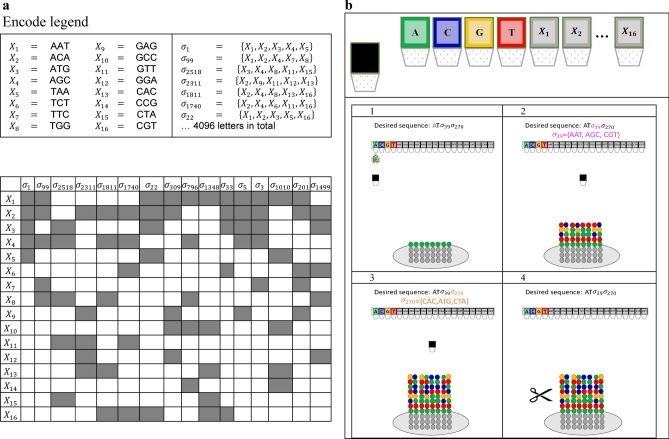
Our combinatorial encoding and synthesis approach. (**a**) Schematic view of a combinatorial alphabet (Encode legend). A set of 16 trimers, ${{\varvec{X}}}_{1},\dots ,{{\varvec{X}}}_{16}$, is used to construct 4096 combinatorial letters, each representing a subset of 5 trimers as indicated on the right and depicted in the grayed-out cells of the table. (**b**) A suggested approach for combinatorial shortmer synthesis. A modified synthesizer would include designated containers for the 16-trimer building blocks and a mixing chamber. Standard DNA synthesis is used for the barcode sequence (1), while the combinatorial synthesis proceeds as follows: The trimers included in the synthesized combinatorial letter are injected into the mixing chamber and introduced into the elongating molecules (2). The process repeats for the next combinatorial letter (3), and finally, the resulting molecules are cleaved and collected (4).

We define a combinatorial alphabet $\Sigma$ over $\Omega$ as follows. Each letter in the alphabet represents a non-empty subset of the elements in $\Omega$. Formally, a letter $\sigma \in \Sigma$, representing a subset $S\subseteq \Omega /\varnothing$, can be written as an N-dimensional binary vector where the indices for which ${\sigma }_{i}=1$ represents the k-mers from $\Omega$ included in the subset S. We denote the k-mers in $S$ as *member k-mers* of the letter $\sigma$. For example, $\sigma =(\mathrm{0,1},\mathrm{0,1},\mathrm{1,0})$ represents $S=\{{X}_{2},{X}_{4},{X}_{5}\}$ and $\left|\Omega \right|=N=6$. Figure 1a,b illustrate an example of a combinatorial alphabet using $N=16$, in which every letter represents a subset of size 5 of $\Omega$. In Sect. “Binary and binomial combinatorial alphabets” includes a description of the construction of different combinatorial alphabets.

To write a combinatorial letter $\sigma$ in a specific position, a mixture of the member k-mers of $\sigma$ is synthesized. To infer a combinatorial letter $\sigma$, a set of reads needs to be analyzed to determine which k-mers are observed in the analyzed position (See Sects. “Binary and binomial combinatorial alphabets” and “Reconstruction probabilities for binomial encoding” for more details). This set of k-mers observed in the sequencing readout and used for inferring $\sigma$ is referred to as *inferred member k-mers.* While the synthesis output and the sequencing readout will include different counts for the member k-mers, the determination of the set of inferred k-mers will force binary assignment for each k-mer to fit into the design scheme of combinatorial encoding.

From a hardware/chemistry perspective, the combinatorial shortmer encoding scheme can potentially be based on using the standard phosphoramidite chemistry synthesis technology, with some alterations (See Fig. 1b and Supplementary Sect. 8.1)^15,16^. First, DNA k-mers should be used as building blocks for the synthesis^17^. Such reagents are commercially available for DNA trimers and were used, for example, for the synthesis of codon optimization DNA libraries^18,19^. In addition, a mixing step should be added to each cycle of the DNA synthesis to allow mixing of the member k-mers prior to their introduction to the elongating molecules. Such systems are yet to be developed and current attempts for combinatorial DNA synthesis are based on enzymatic assembly of longer DNA fragments^13,14^.

Similar to composite DNA encoding, combinatorial encoding requires the barcoding of the sequences using unique barcodes composed of standard DNA barcodes. This design enables direct grouping of reads pertaining to the same combinatorial sequence. These groups of reads are the input for the process of reconstructing the combinatorial letters.

The extended combinatorial alphabets allow for a higher logical density of the DNA-based storage system, while the binary nature of the alphabet minimizes error rates.

### Binary and binomial combinatorial alphabets

The main parameter that defines a combinatorial encoding scheme is the alphabet $\Sigma$. More specifically, it is the set of valid subsets of $\Omega$ that can be used as letters. We define two general approaches for the construction of $\Sigma$. Namely, the *binomial encoding* and the *full binary encoding*.

In the *binomial encoding* scheme, only subsets of $\Omega$ of size exactly $K$ represent valid letters in $\Sigma$, so that every letter $\sigma \in \Sigma$ consists of exactly $K$ member k-mers. Therefore, all the letters in the alphabet have the same Hamming weight $K$. $w\left(\sigma \right)=K, \forall \sigma \in \Sigma$. This yields an effective alphabet of size $$\left|\Sigma \right|=\left(\begin{array}{c}N\\ K\end{array}\right)$$ letters, where each combinatorial letter encodes $${{\text{log}}}_{2}\left(\left|\Sigma \right|\right)={{\text{log}}}_{2}\left(\begin{array}{c}N\\ K\end{array}\right)$$ bits. An r-bit binary message requires $$\frac{r}{{{\text{log}}}_{2}\left(\begin{array}{c}N\\ K\end{array}\right)}$$ synthesis cycles (and a DNA molecular segment with length $$\frac{kr}{{{\text{log}}}_{2}\left(\begin{array}{c}N\\ K\end{array}\right)}$$ ). In practice, we would prefer working with alphabet sizes that are powers of two, where each letter will encode for $$\left\lfloor {\log_{2} \left( {\begin{array}{*{20}c} N \\ K \\ \end{array} } \right)} \right\rfloor$$ bits. Note that this calculation ignores error correction redundancy, random access primers, and barcodes, which are all required for message reconstruction. See Supplementary Sect. 8.2 and Fig. 1a, which illustrate a trimer-based binomial alphabet with $N=16$ and $K=5$, resulting in an alphabet of size $$\left|\Sigma \right|=\left(\begin{array}{c}16 \\ 5 \end{array}\right)=\mathrm{4,368}$$ that allows to encode $\lfloor {log}_{2}(4368)\rfloor =12$ bits per letter or synthesis position.

In the *full binary encoding* scheme, all possible nonempty subsets of $\Omega$ represent valid letters in the alphabet. This yields an effective alphabet of size $\left|\Sigma \right|={2}^{N}-1$ letters, each encoding for $\left\lfloor {\log_{2} \left( {\left| \Sigma \right|} \right)} \right\rfloor = N - 1$ bits.

From this point on, we focus on the binomial encoding.

### Reconstruction probabilities for binomial encoding

In this section, the performance characteristics of binomial encoding are investigated. Specifically, we present a mathematical analysis of the probability of successfully reconstructing the intended message. In Sects. “An end-to-end combinatorial shortmer storage system” and "Experimental proof of concept", results are presented from our simulations and a small-scale molecular implementation of the binomial encoding, respectively.

#### Reconstruction of a single combinatorial letter

Since every letter $\sigma \in \Sigma$ consists exactly of the $K$ member k-mers, the required number of reads for observing at least one read of each member k-mer in a single letter follows the coupon collector distribution^20^. The number of reads required to achieve this goal can be described as a random variable $R={\sum }_{i=1}^{K}{R}_{i}$, where ${R}_{1}=1$ and ${R}_{i}\sim Geom\left(\frac{K-i+1}{K}\right), i=2,\dots ,K$. Hence, the expected number of required reads, is:

1 $$E\left[R\right]={\sum }_{i=1}^{K}E\left[{R}_{i}\right]=K{\sum }_{i=1}^{K}\frac{1}{i}=KHar(K)$$

where $Har(\cdot )$ is the harmonic number.

The expected number of reads required for reconstructing a single combinatorial letter thus remains reasonable for the relevant values of $K$. For example, when using a binomial encoding with $K=5$ the expected number of reads required for reconstructing a single combinatorial letter is roughly $11.5$, which is very close to the experimental results presented in Sect. "Experimental proof of concept".

By Chebyshev’s inequality (See Sect. "Reconstruction probability of a binomial encoding letter"), we can derive a (loose) upper bound on the probability of requiring more than $E\left[R\right]+cK$ reads to observe at least one read of each member k-mer, where $c>1$ is a parameter:

2 $$P\left(\left|R-KHar(K)\right|\ge cK\right)\le \frac{{\pi }^{2}}{6{c}^{2}}$$

For example, when using a binomial encoding with $K=5$, the probability of requiring more than $26.5$ reads (corresponding to $c=3$) is bounded by $0.18$, which is consistent with the experimental result shown in Fig. 5d.

#### Reconstruction of a combinatorial sequence

When we examine an entire $K$-subset binomial encoded combinatorial sequence of length $l$, we denote by $R(l)$ the required number of reads to observe $K$ distinct k-mers in every position. Assuming independence between different positions and not taking errors into account, we get the following relationship between $c$ and any desired confidence level $1-\delta$ (See Sect. "Reconstruction probability of a binomial encoding letter" for details):

3 $$P\left(\left|R\left(l\right)-KHar\left(K\right)\right|\ge cK\right)\le 1-{\left(1-\frac{{\pi }^{2}}{6{c}^{2}}\right)}^{l}<\delta$$

And therefore:

4 $$P\left(R\left(l\right)<KHar\left(K\right)+cK\right)\ge {\left(1-\frac{{\pi }^{2}}{6{c}^{2}}\right)}^{l}\ge 1-\delta$$

The number of reads required to guarantee reconstruction of a binomial encoded message, at a $1-\delta$ probability, with $K=5,$ and $l$ synthesized positions, is thus $KHar\left(K\right)+cK$ when

5 $$c\ge \sqrt{1/6}\pi {\left(1-{\left(1-\delta \right)}^{1/l}\right)}^{-1/2}$$

Supplementary Table S2 shows several examples of this upper bound. As demonstrated in the simulations and the experimental results, this bound is not tight (See Sects. “An end-to-end combinatorial shortmer storage system” and "Experimental proof of concept").

Note that with an online sequencing technology (such as nanopore sequencing) the sequencing reaction can be stopped after $K$ distinct k-mers are confidently observed.

To take into account the probability of observing a k-mer that is not included in $\Omega$ (e.g., due to synthesis or sequencing error), we can require that at least $t>1$ reads of each of the $K$ distinct k-mers will be observed. This is experimentally examined in Sect. "Experimental proof of concept", while the formal derivation of the number of required reads is not as trivial, and will be addressed in future work.

The above analysis is based only on oligo recovery, which depends solely on the sampling rate, ignoring possible mix-up errors (i.e., incorrect k-mer readings). This assumption is based on the near-zero mix-up probability attained by the construction of $\Omega$, which maximizes the minimal Hamming distance between elements in $\Omega$. In Sect. "Experimental proof of concept", this analysis is compared to experimental results obtained from using synthetic combinatorial DNA.

### An end-to-end combinatorial shortmer storage system

**Figure 2:**
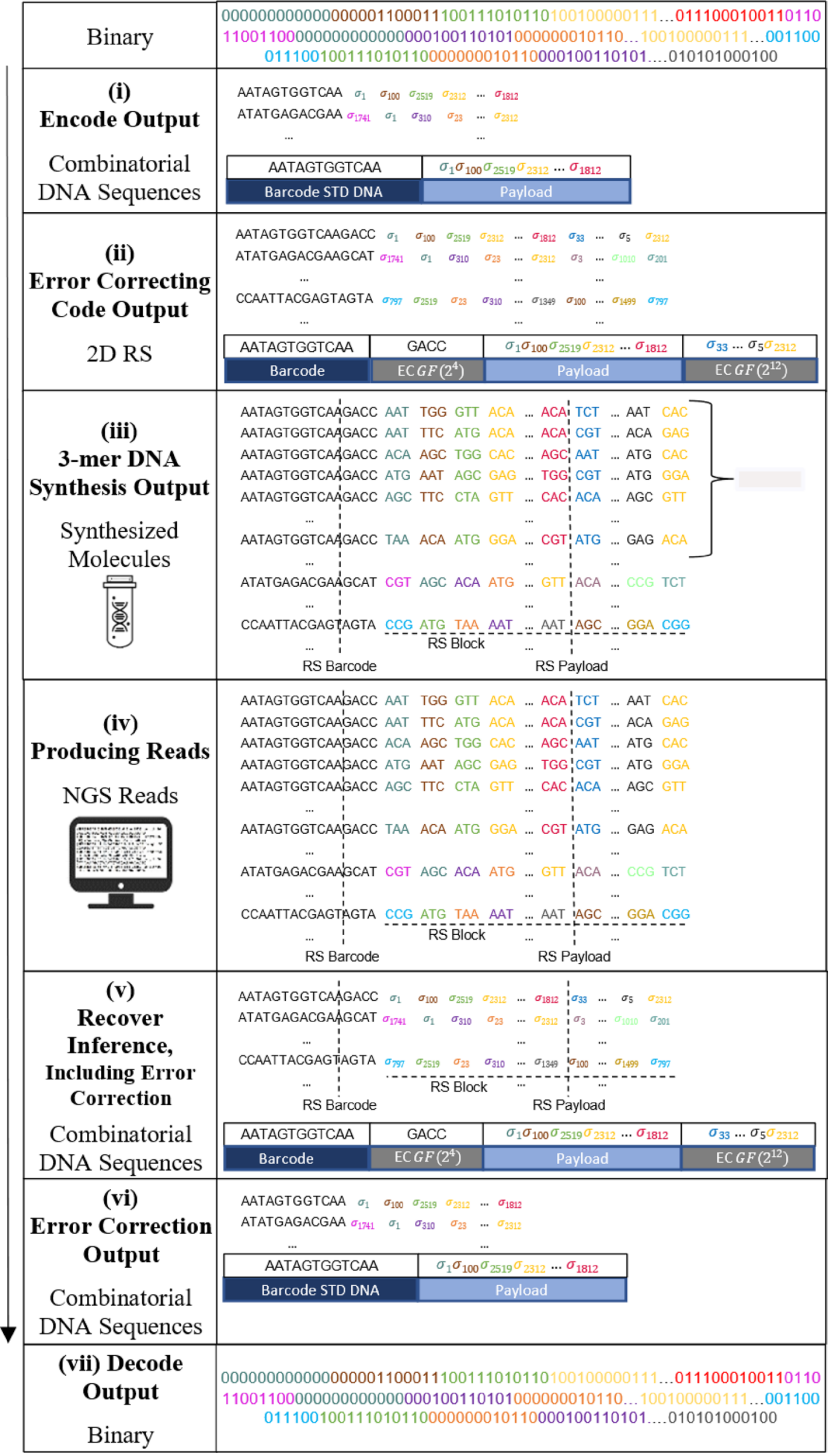
End-to-end workflow of a combinatorial DNA storage system. A binary message is broken into chunks, barcoded, and encoded into a combinatorial alphabet (i). RS encoding is added to each chunk and each column (ii). The combinatorial message is synthesized using combinatorial shormer synthesis (iii), and the DNA is sequenced (iv). Next, the combinatorial letters are reconstructed (v). Finally the message goes through 2D RS decoding (vi), followed by its translation back into the binary message (vii).

We suggest a complete end-to-end workflow for DNA-based data storage with the combinatorial shortmer encoding presented in Fig. 2. The workflow begins with encoding, followed by DNA synthesis, storage, and sequencing, and culminates in a final decoding step. A 2D Reed-Solomon (RS) error correction scheme, which corrects errors in the letter reconstruction (for example, due to synthesis, sequencing, and sampling errors) and any missing sequences (such as dropout errors), ensures the integrity of the system. Table 1 shows the encoding capacities of the proposed system, calculated on a 1 GB input file with standard encoding and three different binomial alphabets (See Supplementary Sect. 8.5). All calculations are based on error correction parameters similar to those previously described (See Sect. "Information capacities for selected encodings")^3,4,21,22^. With these different alphabets, up to a 6.5-fold increase in information capacity is achieved per synthesis cycle, compared to standard DNA-based data storage. While different error correction codes can be used in this system, for our work we chose to implement a 2D RS.

**Table 1:** Logical densities for selected encoding schemes.

Type	$N$	$K$	$$\left(\begin{array}{c}N \\ K\end{array}\right)$$	Bits per letter	Alphabet size	Bits per sequence	Number of sequences	Reed Solomon (RS)	Bits per synthesis cycle, payload only	Bits per synthesis cycle	Fold increase
Standard				2	4	240	33,333,334	38,095,248	1.57	1.40	1.0
Binomial	16	3	560	9	512	1,080	7,407,408	8,465,616	7.05	6.30	4.5
Binomial	16	5	4,368	12	4,096	1,440	5,555,556	6,349,248	9.40	8.40	6.0
Binomial	16	7	11,440	13	8,192	1,560	5,128,206	5,860,848	10.19	9.10	6.5

The numbers represent encoding a 1 GB binary message using oligos with 14nt barcodes + 2nt RS (standard DNA), and 120 payload letters (from $\Sigma$) with 14 extra RS for the payload (the payload and its RS is combinatorial with $N$ and $K$ as indicated).

An example of the proposed approach, using a binomial alphabet with $N=16$ and $K=5$ and 2D RS, is detailed below. A binary message is encoded into a combinatorial message using the 4096-letter alphabet. Next, the message is broken into 120-letter chunks, and each chunk is barcoded. The 12nt barcodes are encoded using RS(6,8) over $GF({2}^{4})$, resulting in 16nt barcodes. Each chunk of 120 combinatorial letters is encoded using RS(120,134) over $GF({2}^{12})$. Every block of 42 sequences is then encoded using RS(42,48) over $GF\left({2}^{12}\right)$ (See Sect. "An end-to-end combinatorial storage system" for details).

**Figure 3:**
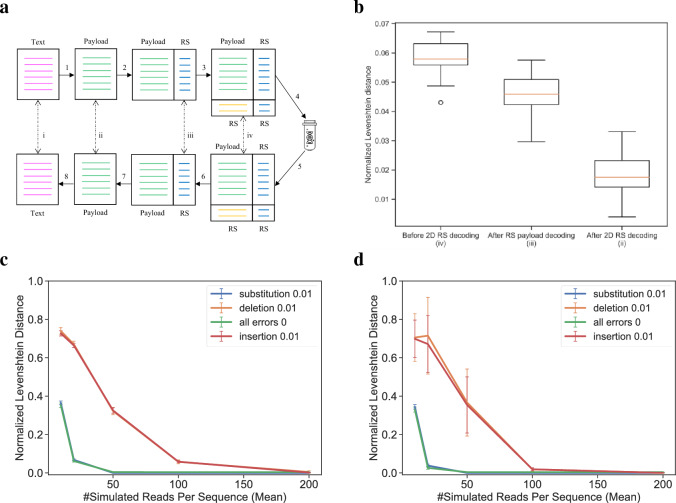
Simulation of an end-to-end combinatorial shortmer encoding. (**a**) A schematic view of the simulation workflow. A text message is translated into a combinatorial message (1), and encoded using RS error correction on the barcode and payload (2). Each block is encoded using outer RS error correction (3). DNA synthesis and sequencing are simulated under various error schemes, and the combinatorial letters are reconstructed (4–5). RS decoding is performed on each block (6) and on each sequence (7) before translation back to text (8). The Roman numerals (i-iv) represent the different error calculations. (**b**) Error rates in different stages of the decoding process. Boxplot of the normalized Levenshtein distance (See Sect. "Reconstruction") for the different stages in a simulation (30 runs) of sampling 100 reads, with an insertion error rate of 0.01. The X-axis represents the stages of error correction (before 2D RS decoding (iv), after RS payload decoding (iii), and after 2D RS decoding (ii)). (**c**,**d**) Sampling rate effect on overall performance. Normalized Levenshtein distance as a function of sampling rate before RS decoding (**c**) and after 2d RS decoding (ii). Different lines represent different error types (substitution, deletion, and insertion) introduced at a rate of 0.01.

To better characterize the potential of this proposed system, we implemented an end-to-end simulation using the parameters mentioned above. We simulated the encoding and decoding of 10 KB messages with different binomial alphabets and error probabilities, and then measured the resulting reconstruction and decoding rates throughout the process. Figure 3a depicts a schematic representation of our simulation workflow and indicates how the error rates are calculated (See Sect. "Reconstruction").

The results of the simulation runs are summarized in Fig. 3b–d. Each run included 30 repeats with random input texts of 10 KB encoded using 98 combinatorial sequences, each composed of 134 combinatorial letters and 16nt barcode, as described above. Each run simulated the synthesis of 1000 molecules on average per combinatorial sequence and sampling of a subset of these molecules to be sequenced. The subset size was drawn randomly from $N\left(\mu ,\sigma =100\right),$ where $\mu$ is a parameter. Errors in predetermined rates were introduced during the simulation of both DNA synthesis and sequencing, as expected in actual usage^23^ (See Sect. "Synthesis and sequencing simulation with errors" for details on the simulation runs). Reconstruction rates and Levenshtein distances are calculated throughout the simulation process, as described in Fig. 3a.

Notably, the sampling rate is the dominant factor where even with zero synthesis and sequencing errors, low sampling rates yield such poor results (Fig. 3c) that the RS error correction is unable to overcome (Fig. 3d). The effect of substitution errors on the overall performance is smaller and they are also easier to detect and correct. This is because substitution errors occur at the nucleotide level rather than at the trimer level. The minimal Hamming distance $d=2$ of the trimer set $\Omega$ allows for the correction of single-base substitutions. The use of 2D RS error correction significantly improved reconstruction rates, as can be observed in Fig. 3b.

**Figure 4:**
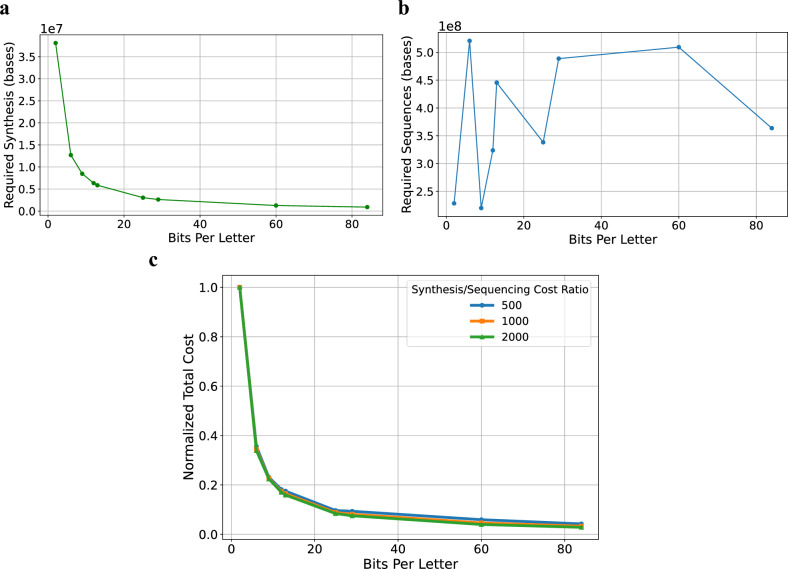
Cost analysis for a combinatorial DNA-based data storage system using different alphabets. (**a**) synthesis cost as a function of the alphabet size (presented as bit per letter, for simplicity). The cost is calculated as the number of synthesis cycles required for storing 1 GB of information. (**b**) Sequencing cost as a function of the alphabet size. Similarly to (**a**). (**c**) Normalized total cost as a function of the alphabet size for different synthesis-to-sequencing cost ratios. Costs are normalized by the total cost of a standard DNA-based system.

To assess the effect of using the suggested approach on the cost of DNA-based data storage systems, we performed an analysis of the different cost components. In brief, we analyzed the effect on the number of synthesis cycles and the number of bases to sequence, taking into account the required sequencing depth to achieve a desired reconstruction probability (See Sect. "Cost analysis"). Figure 4 depicts the costs of storing 1 GB of information using different combinatorial alphabets. Clearly, combinatorial DNA encoding can potentially reduce DNA-based data storage costs as the alphabet size grows and each letter encodes more bits. This is especially relevant in comparison with the composite encoding scheme presented in^3^. While both methods increase the logical density by extending the alphabet using mixtures of DNA letters/k-mers and thus reducing the synthesis cost (See Fig. 4a), a crucial difference lies in the effect on sequencing costs. Composite DNA uses mixed letters with varying proportions of the different letters, which makes reconstruction very challenging in larger alphabets and results in very high sequencing costs that undermine the reduced synthesis costs. On the other hand, combinatorial DNA encoding uses binary mixtures, which are much simpler to reconstruct, therefore maintaining the sequencing costs relatively constant as the alphabet grows (See Fig. 4b). For assessing the sequencing costs, we used a coupon collector model presented in^24^ to calculate the required sequencing depth that ensures a decoding probability with an error rate of less than ${10}^{-4}$ (See Supplementary Sect. 8.5). In comparison with the composite encoding scheme, our analysis demonstrates a required sequencing depth that grows moderately. Figure 4c analyzes the normalized overall cost, based on different assumptions regarding the ratio between synthesis costs and sequencing costs, ${C}_{syn}:{C}_{seq}$. With a cost ratio of 500:1, 1000:1, 2000:1, it is evident that synthesis costs outweigh the fluctuations in sequencing costs, indicating a monotonic reduction in overall costs. This is an improvement compared to the composite DNA approach presented in^3^, where costs are reduced only up to a certain alphabet size, and then increase again due to the increased sequencing cost. In combinatorial DNA encoding, costs continue to drop, while alphabet size increases.

### Experimental proof of concept

**Figure 5:**
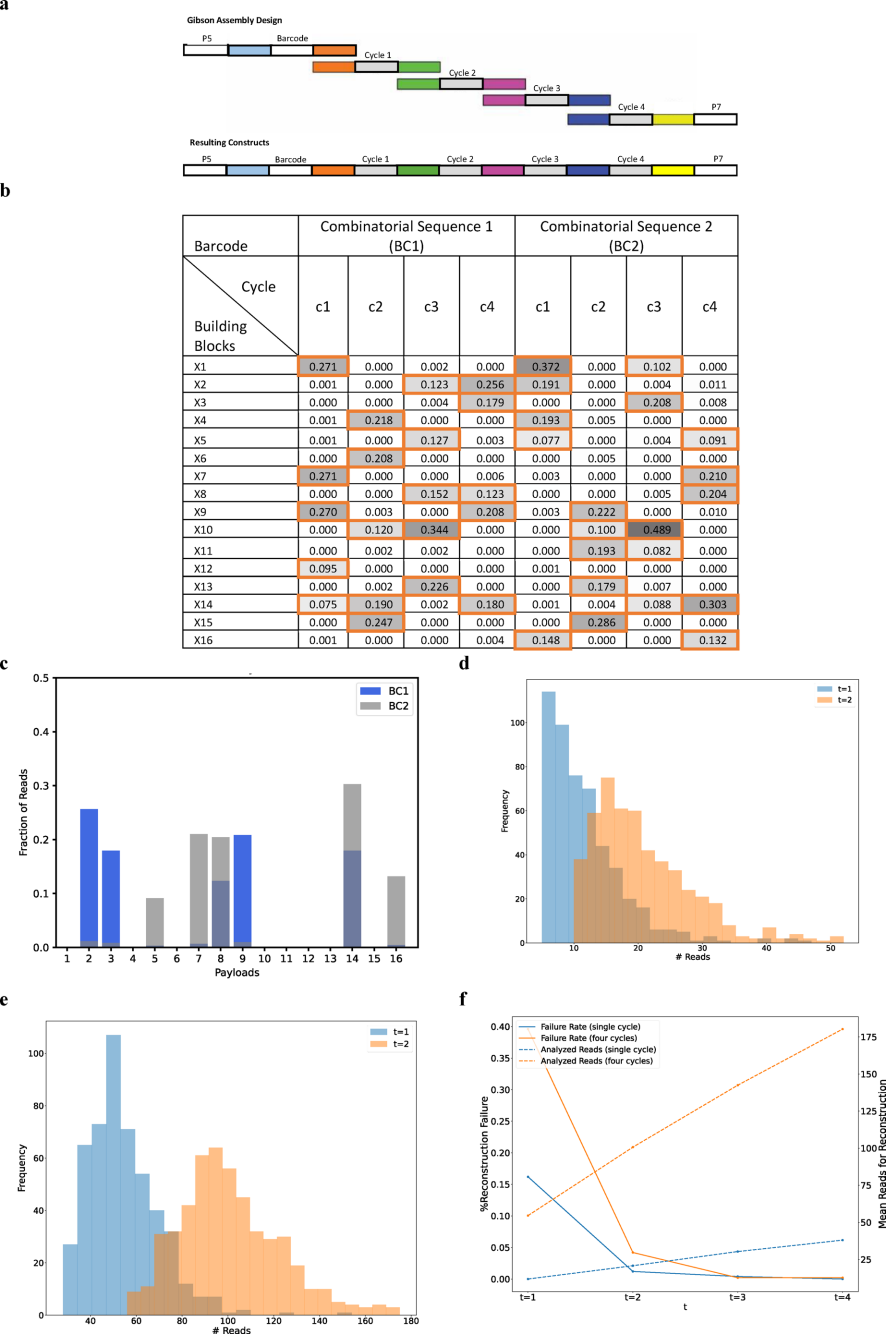
Experiment analysis. (**a**) A schematic view of the Gibson assembly. Each combinatorial sequence consists of a barcode segment and four payload segments (denoted as cycles 1–4). (**b**) Reconstruction results of the two combinatorial sequences. The color indicates read frequency, and the member k-mers are marked with orange boxes. (**c**) The distribution of reads over the 16 k-mers in an example combinatorial letter. Overlaid histograms represent the percentage of reads for each of the 16 k-mers for the same position in our two combinatorial sequences. This, in fact, is an enlarged view of the two c4 columns of panel b. (**d**) Required number of reads for reconstructing a single combinatorial letter. A histogram of the number of reads required to observe at least $t=\mathrm{1,2}$ reads from $K=5$ inferred k-mers. The results are based on resampling the reads 500 times, the data represents cycle 4. (**e**) Required number of reads for reconstructing a four-letter combinatorial sequence. Similar to d. (**f**), Reconstruction failure rate as a function of the required multiplicity $t$. Erroenous reconstruction rate shown for different values of required copies to observe each inferred k-mer ($t=\mathrm{1,2},\mathrm{3,4}$). The mean required number of reads for reconstruction is displayed using a secondary Y-axis in the dashed lines.

To assess and establish the potential of large combinatorial alphabets, we performed a small-scale experimental proof of concept study demonstrating the encoding and decoding of a 96-bit input message, which is equivalent to the text “DNA Storage!”. Since combinatorial DNA synthesis technology is not yet available, we demonstrated the combinatorial approach using Gibson assembly as an ad-hoc imitation for combinatorial synthesis. We constructed two combinatorial sequences, each containing a barcode and four payload cycles over a binomial alphabet with $N=16$ and $K=5$. The assembly was performed using DNA fragments composed of a 20-mer information sequence and an overlap of 20 bp between adjacent fragments, as shown in Fig. 5a. The assembled DNA was then stored and sequenced for analysis using Illumina Miseq (See Table 3 and Sect. "Cost analysis" for details about the sequencing procedures).

The sequencing output was then analyzed using the procedure described in Sect. “Decoding and analysis”. Both combinatorial sequences were successfully reconstructed from the sequencing reads, as presented in Fig. 5b, and Supplementary Figs. S1, S2, and S3. The experiment also demonstrated the robustness of the binomial DNA encoding for synthesis and sequencing errors, as described in Fig. 5c. We observed a minor leakage between the two synthesized sequences, which was overcome by the reconstruction pipeline (See Fig. 5c, and Supplementary Figs. S1, S2, and S3). Note that there is a partial overlap between the member k-mers of the two sequences.

For comparison, a recent study by^14^ encoded the 84-bit phrase “HelloWord” using a different encoding and synthesis approach. A comparison between the two experiments is shown in Table 2. For example, while we used Gibson assembly as our synthesis method, they introduced a new method called Bridge Oligonucleotide Assembly. We encoded 12 bits per synthesis cycle and assembled four combinatorial fragments in each sequence, while^14^ encoded 84 bits in a single combinatorial cycle. Our 96-bit were split and encoded using two combinatorial sequences, while they encoded the same 84-bits message, in its full format, on eight different sequences repeatedly. Our ${\text{N}}=16$ and 5 combinatorial factor, while their $N=96$ and a higher 32 combinatorial factor.

**Table 2:** Comparison of our experiment with data from^14^.

	This work	Yan et al.^14^
Message length (bits)	96	84
Logical density (bits/ Synthesis Cycle)	12	84
Sequence length (Cycles)	4	1
Number of sequences	2	8
Number of unique k-mers (N)	16	96
Number of unique k-mers in each letter (K)	5	32
Sequence length	220 bp	75 bp
Barcode length	20 bp	25 bp
Space / Adjacent fragments	20 bp	25 bp, same in all BC
Payload length	20 bp	25 bp
Sequencing method	Ilumina Miseq	Nanopore
Synthesis	Gibson Assembly	Bridge Oligonucleotide Assembly
Amount of space / Adjacent fragments	6	1

To test the effect of random sampling on the reconstruction of combinatorial sequences, we performed a subsampling experiment with $N=500$ repeats, presented in Fig. 5d–f. We subsampled varying numbers of reads from the overall read pool and ran the reconstruction pipeline. Note that, as explained, the reconstruction of a single binomial position requires finding $K=5$ inferred k-mers. That is, observing five unique k-mers at least $t$ times. We tested the reconstruction performance using $t=\mathrm{1,2},\mathrm{3,4}$ and recorded the effect on the successful reconstruction rate and required number of reads.

For $t=1$, reconstruction required analyzing 12.26 reads on average. These included 0.45 reads that contained an erroneous sequence that could not be mapped to a valid k-mer, and thus ignored. Note that the design of the set $\Omega$ of valid k-mers allows us to ignore only the reads for which the Hamming distance for a valid k-mer exceeded a predefined threshold ($d=3$). If we ignored all the reads containing a sequence with non-zero Hamming distance to all k-mers, we would have skipped 2.26 extra reads, on average.

As expected, requiring $t=2$ copies of each inferred k-mer resulted in an increase in the overall number of analyzed reads. Reconstruction of a single combinatorial letter required analyzing an average of 21.6 reads with 0.83 skipped and 3.99 non-zero Hamming distance reads. The complete distribution of the number of reads required for the reconstruction of a single position using $t=\mathrm{1,2}$ is presented as a histogram in Fig. 5d.

To reconstruct a complete combinatorial sequence of 4 positions, we required the condition to hold for all positions. For $t=1$, this entailed the analysis of 55.60 reads on average, out of which 1.04 reads were identified as erroneous and thus ignored, and with 7.36 non-zero Hamming distance reads. For $t=2$, an average of 102.66 reads were analyzed with 1.97 skipped and 13.24 non-zero Hamming distance reads. The complete distribution of the number of reads required for reconstructing a complete combinatorial sequence using $t=\mathrm{1,2}$ is presented as a histogram in Fig. 5e.

Note that these results correspond to the analysis presented in Sect. “Reconstruction probabilities for binomial encoding”, for the reconstruction of a single binomial position and a complete binomial sequence. Calculating the bound presented in Supplementary Table S2, with $K=5$ and $l=4$, yields a requirement of approximately 140 reads to obtain $1-\delta =0.99$ probability of reconstruction. Clearly, this is well above the observed number of 55.60 reads. Note, as explained, the calculated bound is a loose bound.

The reconstruction procedure ends with a set of inferred k-mers that represent the inferred combinatorial letter. This set is not guaranteed to be correct, especially when using $t=1$, which means that noisy reads may result in an incorrect k-mer included in the inferred letter. Figure 5f depicts the rate of incorrect reconstructions as a function of the number of required copies for each inferred k-mer ($t=\mathrm{1,2},\mathrm{3,4}$). Note that with $t\ge 3$ results in 100% successful reconstruction. This, however, comes with a price, where more reads must be analyzed.

## Discussion

In this study, we introduced combinatorial shortmer encoding for DNA-based data storage, which extends the approach of composite DNA by resolving some of its sensitivity related issues. Combinatorial shortmer encoding allows for increased logical density while maintaining low error rates and high reconstruction rates. We explored two encoding schemes, binary and binomial, and evaluated some of their theoretical and practical characteristics. The inherent consistency of the binomial encoding scheme, where every letter in the sequence consists of exactly $K$ distinct member k-mers, ensures uniformity in the encoded DNA sequences. This approach not only simplifies the reading process, but also allows for a more streamlined decoding. For instance, technologies like nanopore sequencing enable continuous sequencing until all k-mers at a given position are confidently observed.

Our suggested approach is designed to inherently overcome base substitution errors, which are the most common errors expected in every DNA-based data storage system that includes DNA sequencing. This is achieved by the selection of a set of $N$ k-mer building blocks to be resilient to single-base substitutions. Other considerations may also be incorporated in the selection of the set of valid k-mers, taking into account any biological, chemical, or technological constraints. This represents an inherent tradeoff in DNA-based data storage between sequence constraints and information density. Insertion and deletion errors, which usually originate in the synthesis process, are more challenging to overcome. We introduced a 2D RS error correction scheme on the shortmer level, allowing for a successful message reconstruction even with error levels exceeding those expected in reality.

Our study highlights the significant effect of sampling rates on the overall performance of the system. The accuracy and completeness of sequence reconstruction require each of the sequences to be observed with a sufficiently high coverage. Our subsampling experiments underpin this observation, demonstrating the need for calibration of sampling rates to ensure the desired fidelity in DNA-based data storage. The crucial role of the sampling rate was also highlighted in^3^. However, while composite DNA uses mixed letter with varying proportions of the different letters, the combinatorial encoding, studied in this current work, uses binary mixtures and does not rely on proportions. This potentially allows scaling up the combinatorial encoding without a significant effects on the required sampling rates.

Combinatorial DNA coding can potentially reduce the overall costs of DNA-based data storage. Considering both sequencing costs, which fluctuate, and synthesis costs, which consistently drop, the increase in the alphabet size is accompanied by a decrease in overall cost. However, combinatorial DNA synthesis or assembly is still unavailable for large-scale commercial use. Thus, further development of combinatorial DNA synthesis technologies will continue to impose limitations and constraints on combinatorial encoding, and determine the overall costs.

While our proof-of-concept experiment showed success on a small scale, there are complexities to be addressed in considering large-scale applications. These include synthesis efficiency, error correction, and decoding efficiency. Nonetheless, the resilience of our binomial DNA encoding for both synthesis and sequencing errors highlights its practical potential and scalability. One specific aspect is the effect of combinatorial encoding on possible sequence-related constraints. While sequences with unwanted compositions (e.g., containing homopolymers) will unavoidably be part of the synthesized mixtures, the uniform sampling of the combinatorial shortmers in each position, together with the independence of the different positions, guarantees that only very few such sequences will be aythesized. In particular—these will not interfere with successful reconstruction. Another challenging aspect of scaling up combinatorial DNA systems is the need to use longer DNA k-mers to construct larger sets with the desired constraints. This may make the combinatorial synthesis impractical and will require balancing the increase in logical density with the technological complexity.

Several future research directions emerge from our study. First, it is important to develop error correction methods for better handling insertion and deletion errors. One approach for achieving this goal, is to adjust sampling rates: optimizing the sampling rate, especially in large-scale experiments, can lead to data retrieval at high accuracy. While our study highlighted the role of sampling rates in achieving desired outcomes, delving deeper into the underlying theory will lead to more improvements. For example, based on theoretical bounds of sampling rates, more concrete recommendations can be provided for real-world applications. The development of error correction codes, designed specifically to overcome the error types that characterize combinatorial encoding, is another important direction for future research. Most notably, transitioning from small-scale proof-of-concept experiments to larger-scale implementations is an important next step. Evaluating the scalability of our method across various scales and complexities will be enlightening, especially when considering synthesis efficiency and error rates. Finally, the consideration of advanced sequencing technologies could redefine the potential and efficacy of our proposed method, including its future practical implementation.

To sum up, combinatorial DNA synthesis and sequence design are important beyond the scope of DNA-based data storage. Generating combinatorial DNA libraries is an efficient tool in synthetic biology, better supporting large-scale experiments. DNA synthesis technologies that can incorporate a combinatorial synthesis of longer DNA fragments will enable the design and generation of more DNA libraries with applications in data storage and beyond.

## Methods

### Reconstruction probability of a binomial encoding letter

Let the number of reads required for reconstruction be a random variable $R={\sum }_{i=1}^{K}{R}_{i}$ where ${R}_{1}=1$ and ${R}_{i}\sim Geom\left(\frac{K-i+1}{K}\right), i=2,\dots ,K$. Hence, the expected number of required reads, is:

6 $$E\left[R\right]={\sum }_{i=1}^{K}E\left[{R}_{i}\right]=K{\sum }_{i=1}^{K}\frac{1}{i}=KHar(K)$$

where $Har(\cdot )$ is the harmonic number.

Using the independence of ${R}_{i}$, the variance of $R$ can be bound by (See^25^):

7 $$Var\left(R\right)={\sum }_{i=1}^{K}Var\left({R}_{i}\right)<{K}^{2}\left(\frac{1}{{1}^{2}}+\frac{1}{{2}^{2}}+\dots +\frac{1}{{K}^{2}}\right)<\frac{{\pi }^{2}}{6}{K}^{2}$$

By Chebyshev’s inequality, we get an upper bound (a loose bound) on the probability of requiring more than $E\left[R\right]+cK$ reads to observe at least one read of each member k-mer:

8 $$P\left(\left|R-E\left(R\right)\right|\ge b\sigma \right)\le \frac{1}{{b}^{2}}$$

9 $$P\left(\left|R-E\left(R\right)\right|\ge b\frac{\pi }{\sqrt{6}}K\right)\le \frac{1}{{b}^{2}}$$

Let $c=b\frac{\pi }{\sqrt{6}}$, or $b=\frac{c\sqrt{6}}{\pi }$, and we obtain:

10 $$P\left(\left|R-E\left[R\right]\right|\ge cK\right)\le \frac{{\pi }^{2}}{6{c}^{2}}$$

Or specifically:

11 $$P\left(\left|R-KHar\left(K\right)\right|\ge cK\right)\le \frac{{\pi }^{2}}{6{c}^{2}}$$

We now turn to address the reconstruction of an entire oligo of length $l$. Let $R(l)$ be the random variable representing the number of reads required to have observed all the $K$ member k-mers in every position. Setting any $\delta >0$, if we show that $P\left(R\left(l\right)>m\right)\ge 1-\delta$, then we know that by accumulating $m$ reads the probability of correct full reconstruction is more than $1-\delta$. From Eq. (11), and assuming independence of the positions (in terms of observing all $K$ member k-mers), we get Eq. (12):

12 $$P\left(R\left(l\right)<KHar\left(K\right)+cK\right)\ge {\left(1-\frac{{\pi }^{2}}{6{c}^{2}}\right)}^{l}$$

From which we can extract $c$, so that:

13 $${\left(1-\frac{{\pi }^{2}}{6{c}^{2}}\right)}^{l}\ge 1-\delta$$

Which yields:

14 $$c\ge \frac{\pi }{\sqrt{6\left(1-{\left(1-\delta \right)}^\frac{1}{l}\right)}}$$

This process allows us to evaluate the sequencing depth complexity. For example, consider $l=100$ and $\delta =0.01$. We want to find $c$, so that using $KHar\left(K\right)+cK$ reads will reconstruct the entire sequence with 0.99 probability. We therefore set:

15 $${\left(1-\frac{{\pi }^{2}}{6{c}^{2}}\right)}^{100}\ge 0.99$$

And get:

16 $${\text{c}}\ge \frac{\pi }{\sqrt{6\left(1-{\left(0.99\right)}^{0.01}\right)}}=127.94$$

And therefore, using 128 reads guarantees reconstruction with 0.99 probability.

### An end-to-end combinatorial storage system

In Sect. “An end-to-end combinatorial shortmer storage system” we propose an end-to-end combinatorial storage system, as follows.

#### Combinatorial encoding and padding

A binary message is encoded using a large k-mer combinatorial alphabet (e.g., trimer-based alphabet of size $\left|\Sigma \right|=4096$ letters, with $N=\left|\Omega \right|=16$), resulting in $r=12$ bits per combinatorial letter. The binary message is zero-padded to ensure its length is divisible by $r$ prior to the combinatorial encoding. The complete message is broken into sequences of set length $l=120$, each sequence is then marked with a standard DNA barcode and translated using the table presented in the Encode legend (See Supplementary Sect. 8.2).

**Figure 6:**
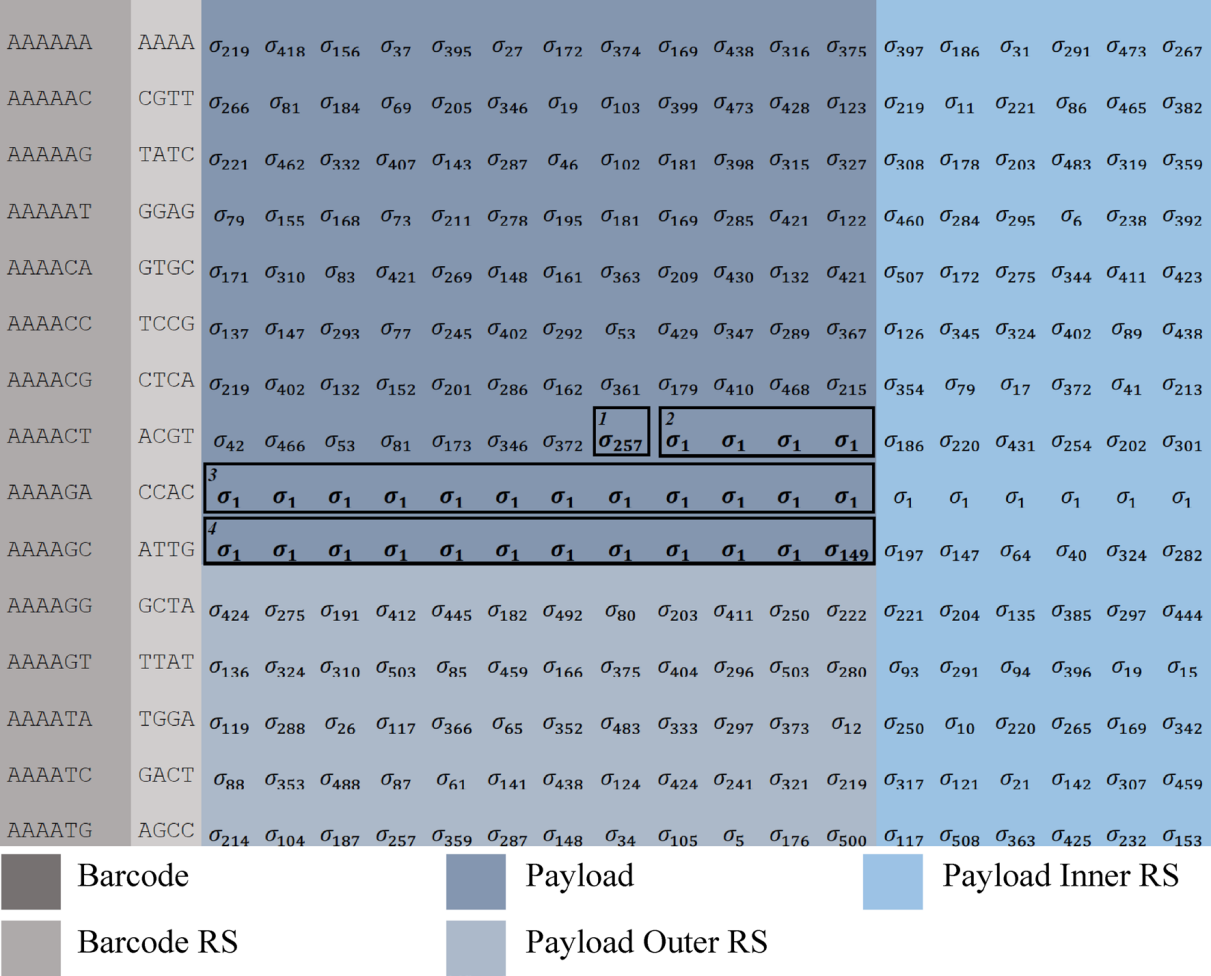
Example of message coding, including padding and RS error correction. Encoding of a ~ 0.1 KB message into a 512 letter binomial alphabet ($$N=16, K=3)$$. First, bit padding is added, included here in the letter $$_{{\sigma_{257} }}^{1}$$. Next, block padding is added, included here in $$_{{\sigma_{1} }}^{2}$$ and $$_{{\sigma_{1} }}^{3}$$. Padding information is included in the last sequence of all blocks. The last sequence holds the number of padding binary bits. In this example, $$_{{\sigma_{149} }}^{4}$$ represents 148 bits of padding, composed of $$4+\left(4*9\right)+\left(12*9\right) bits$$, 4 bits from $$_{{\sigma_{257} }}^{1}$$, 4 letters from $$_{{\sigma_{1} }}^{2}$$ and 12 letters from $$_{{\sigma_{1} }}^{3}$$.

The length of the complete combinatorial sequence must be divisible by the payload size $l$ and by the block size $B$. As described in Fig. 6, this is ensured using another padding step, and the padding information is included in the final combinatorial sequence.

#### Error correction codes

The 2D error correction scheme includes the use of three RS^26^ encodings: on each barcode, on the payload part of each sequence, and an outer error correction code on each block of sequences.

- Each barcode is encoded using a systematic RS(6,8) code over $GF({2}^{4})$, transforming the unique 12nt barcode into a 16nt sequence.
- Each 120 combinatorial letter payload sequence is encoded using an RS(120,134) code over $GF({2}^{12})$, resulting in a sequence of length 134 combinatorial letters.
- To protect against sequence dropouts, an outer error correction code is used on the columns of the matrix (See Fig. 6). Each block of $B=42$ sequences, is encoded using a RS(42,48) RS code $GF\left({2}^{12}\right)$. This is applied in each column separately.

For simplicity, Fig. 6 demonstrates the encoding of ~ 0.1 KB using shorter messages with simpler error correction codes. The following parameters are used:

- A barcode length of 6nt encoded using RS(3,5) code over $GF\left({2}^{4}\right)$ to get 10nt.
- A payload length of $l=12$ encoded using RS(12,18) over $GF\left({2}^{9}\right)$ for the $$\left(\begin{array}{c}16\\ 3\end{array}\right)$$ binomial alphabet.
- A 10-sequence block encoded, column-wise, using a (10,15) RS code over $GF({2}^{9})$.

The 824 bits are first padded to be $828=92*9$. The 92 combinatorial letter message is split into $7$ sequences of 12 letters and an additional sequence of 8 letters. Finally, a complete block of 12 sequences (total of $10*12=120$ letters) is created by padding with one additional sequence of 12 letters and including the padding information as the last sequence.

#### Synthesis and sequencing simulation with errors

- **Simulating the synthesis process.** DNA molecules pertaining to the designed sequences are synthesized using combinatorial k-mer DNA synthesis (See Fig. 1b). For each combinatorial sequence, we first determine the number of synthesized copies by sampling from $X\sim N(\mu =1000, {\sigma }^{2}=100)$. Let $x$ be the number of copies for a specific sequence. Next, for every position in the sequence, we uniformly sample $x$ independent k-mers from the set of member k-mers of the combinatorial letter in the specific position. We concatenate the sampled k-mers to the already existing $x$ synthesized molecules.
- **Error simulation.** Synthesis and sequencing errors are simulated as follows. Error probabilities for deletion, insertion, and substitution are given as parameters denoted as ${P}_{d}, {P}_{I},$ and ${P}_{s}$ respectively. Deletion and insertion errors are assumed to occur during k-mer synthesis and thus implemented on the k-mer level (i.e., an entire k-mer is deleted or inserted in a specific position during the synthesis simulation). Substitution errors are assumed to be sequencing errors and hence implemented on a single base level (i.e., a single letter is substituted, disregarding the position within the k-mer).
- **Mixing.** Post synthesis, molecules undergo mixing to mirror genuine molecular combinations. This is achieved through a randomized data line shuffle using an SQLite database, enabling shuffle processes even for sizable input files^27^.
- **Reading and sampling.** From the simulated synthesized molecule set, a subsample of predefined size $$S*number of synthesized seqeunces$$ is drawn, simulating the sampling effect of the sequencing process.

#### Reconstruction

- **Barcode decoding** The barcode sequence of each read is decoded using the RS(6,8) code.
- **Grouping by barcode** The reads are then grouped by their barcode sequence to allow the reconstruction of the combinatorial sequences.
- **Filtering of read groups** Barcodes (set of reads) with less than 10% of the sampling rate $S$ reads are discarded.
- **Combinatorial reconstruction** For each set of reads, every position is analyzed separately. The $K$ most common k-mers are identified and used to determine the combinatorial letter $\sigma$ in this position. Let $\Delta$ be the difference between the length of the analyzed reads and the length of the designed sequence. $\Delta =l-len(read)$. Reads with $\left|\Delta \right|>k-1$ are discarded from the analysis. Invalid k-mers (not in $\Omega )$ are replaced by a dummy k-mer ${X}_{dummy}$.
- **Missing barcodes** Missing barcodes are replaced with dummy sequences to enable correct outer RS decoding.
- **Normalized Levenshtein distance** Levenshtein distance between the observed sequence $O$ and the expected sequence $E$ is calculated^28,29^. Normalized Levenshtein distance is calculated by dividing the distance by the length of the expected sequence:
  17 $$Normalized\; Levenshtein\; distance\; (O,E)=\frac{Levenshtein\; distance\; \left(O,E\right)}{|O|}$$

#### Cost analysis

Synthesis cost estimation was performed using the logical density calculation presented in Supplementary Sect. 8.5 and Supplementary Table S1. To calculate the sequencing costs, we used the coupon collector model presented in^24^ to assess the required sequencing depth given the combinatorial alphabet. Figure 4b indicates the total number of reads required for reconstructing the sequences, calculated as the required sequencing depth multiplied by the number of sequences from Supplementary Sect. 8.5 and Supplementary Table S1. The analysis was performed on the following set of combinatorial alphabets: Standard DNA, $$\left(\begin{array}{c}8\\ 4\end{array}\right), \left(\begin{array}{c}16\\ 3\end{array}\right), \left(\begin{array}{c}16\\ 5\end{array}\right), \left(\begin{array}{c}16\\ 7\end{array}\right), \left(\begin{array}{c}32\\ 10\end{array}\right), \left(\begin{array}{c}32\\ 16\end{array}\right), \left(\begin{array}{c}64\\ 32\end{array}\right), \left(\begin{array}{c}96\\ 32\end{array}\right)$$.

#### Proof of concept experiment

The proof-of-concept experiment was performed by imitating combinatorial synthesis using Gibson assembly of larger DNA fragments. Each DNA fragment was composed of a 20-mer information sequence and an overlap of 20 bp between adjacent fragments, as depicted in Fig. 5a. Two combinatorial sequences were designed, each composed of a barcode fragment, 4 payload fragments, and Illumina Miseq P5 and P7 anchors at the ends. The information fragments included in each combinatorial position were chosen from a set of 16 sequences with sufficient pair-wise distance. The full list of DNA sequences and the design of combinatorial sequences is listed in Supplementary Sect. 8.6.

#### DNA assembly and sequencing

Payload, barcode, and P7 anchor fragments with 20 bp overlaps for the purpose of Gibson assembly were produced by annealing complementary oligonucleotides manufactured by Integrated DNA Technologies (IDT). Oligos were dissolved in Duplex Buffer (100 mM Potassium Acetate; 30 mM HEPES, pH 7.5; available from IDT) to the final concentration of 100 micromolar. For annealing, 25 µl of each oligo in a pair were combined to the final concentration of 50 micromolars. The oligo mixes were incubated for 2 min at 94^0^ C, and gradually cooled down to room temperature. The annealed payload oligos that belonged to the same cycle (5 oligos total) were mixed to the final concentration of 1 micromolar per oligo—a total of 5 micromolar, by adding 2 µl of each annealed oligo into the 90 µl of nuclease-free water—a final volume of 100 µl. Annealed barcode and P7 anchor oligos were also diluted to the final concentration of 5 micromolar in nuclease-free water, after thorough mixing by vortexing. The diluted oligos were stored at −20 °C.

Immediately prior to the Gibson assembly, payload oligo mixes, barcode, and P7 anchor oligos were further diluted 100-fold to the final working dilution of 0.05 pmol/microliter in nuclease-free water. Gibson reaction was assembled by adding 1 µl (0.05 pmol) of barcode, 4 × cycle mixes, and P7 anchor to the 4 µl of nuclease-free water and supplemented with 10 µl of NEBuilder HiFi DNA assembly master mix (New England Biolabs (NEB)) to the final volume of 20 µl, according to the manufacturer instructions. The reactions were incubated for 1 h at 50 °C, and purified with AmpPure Beads (Thermo Scientific) at 0.8X ratio (16 µl of beads per 20 µl Gibson reaction) to remove free oligos / incomplete assembly products. After adding beads and thorough mixing, the reactions were incubated for 10 min at room temperature and then placed on a magnet for 5 min at room temperature. After removing the sup, the beads were washed twice with 100 µl of 80% ethanol. The remaining washing solution was further removed by a 20 µl tip, and the beads dried for 3 min on the magnet with an open lid. After removing from the magnet, the beads were resuspended in 22 µl of IDTE buffer (IDT), incubated for 5 min at room temperature, and then placed back on the magnet.

20 µl of eluate were transferred into the separate 1.7 ml tube. 5 µl of the eluted DNA were used as a template for PCR amplification combined with 23 µl of nuclease-free water, 1 µl of 20 micromolar indexing primer 5, 1 µl of 20 micromolar indexing primer 7, and 10 µl of rhAMPseq master mix v8.1—a total of 40 µl. After initial denaturation of 3 min at 95 °C, the PCR reaction proceeded with 50 cycles of 15 s at 95 °C, 30 s at 60 °C, and 30 s at 72 °C, followed by final elongation of 1 min at 72 °C and hold at 4 °C. The PCR reactions were purified with Ampure beads at 0.8X ratio (32 µl beads per 40 µl of PCR reaction) as outlined above, and eluted in 22 µl IDTE buffer. The concentration and the average size of the eluted product were determined by Qubit High Sensitivity DNA kit and Agilent 2200 TapeStation system with D1000 high-sensitivity screen tape respectively. The eluted product was diluted to 4 nM concentration, and used as an input for denatured sequencing library preparation, per manufacturer instructions. The sequencing was performed on Illumina Miseq apparatus (V2 chemistry, 2 × 150 bp reads) using 6 picomolar denatured library supplemented with 40% PhiX sequencing control.

#### Decoding and analysis

This section outlines the key steps involved in our sequencing analysis pipeline, aimed at effectively processing and interpreting sequenced reads. The analysis pipeline gets the sequencing output file containing raw reads in “.fastq” format and a design file containing the combinatorial sequences.

Analysis steps:

1. **Length filtering.** We saved reads that were 220 bp in length, retaining only those corresponding to our designed read length.
2. **Read retrieval.** We carefully checked each read for the presence of BCs, universals, and payloads. To keep our data accurate, we discarded reads where the BCs, universals, or payloads had a Hamming distance of more than 3 errors.
3. **Identifying inferred k-mers.** For every BC and each cycle, we counted the $K$ most common k-mers. We then compared these with the design file to quantify those matching (Fig. 5b) (See Table 3).

**Table 3:** Summary of sequencing reads analyzed in the study.

Reads	Count
Total PF reads	2,634,683
Reads of length 220	2,139,071
BC1	1,365,295
BC2	768,755
No BC	5021

The table shows the total number of reads obtained, the number filtered by length (220 bases) for analysis, and the counts of reads associated with BC1, BC2, and those that did not have any recognizable barcode (No BC).

### Information capacities for selected encodings

Table 1 illustrates the logical densities derived from encoding a 1 GB binary message using oligonucleotides with a 12nt barcode and an additional 4nt for standard DNA RS error correction, and a 120 letters payload with 14 extra RS for the payload in combinatorial encoding schemes with parameters $N$ and $K$.

The densities were calculated as follows:

18 $${\text{Bits per Letter }} = \left\lfloor {\log_{2} \left( {\left( {\begin{array}{*{20}c} N \\ k \\ \end{array} } \right)} \right)} \right\rfloor$$

19 $$\mathrm{Alphabet\; Size }={2}^{Bits\; per\; Letter}$$

20 $$\mathrm{Bits\; per\; Sequence }=Bits\; per\; Letter\times Payload\; length.$$

21 $$\mathrm{Number\; of\; Sequences }=\lceil\frac{Message\; Size\; in\; bits}{Bits\; per\; Sequence}\rceil$$

22 $${\text{Number\; of\; sequences\; padded}}:{\text{ Total\; number\; of\; sequences\; after\; padding\; for\; the\; block \; size}}$$

23 $$\mathrm{Padding\; is }=Block\; Size-\left(Number\; of\; Sequences \% Block\; Size\right)$$

24 $$\mathrm{Number\; of\; sequences\; with\; RS}=\frac{Number\; of\; sequences\; padded}{Block\; Size}\times Block\; Size\; After RS$$

25 $$\mathrm{Synthesis\;Cycles }=\mathrm{Number\;of\;sequences\;with\;RS}\times Full\;sequence\;length\;with\;RS$$

26 $$\mathrm{Bits\;per\;Synthesis\;Cycle }=\frac{Message\;Size\;in\;bits}{\mathrm{Synthesis\;Cycle}}$$

27 $$\mathrm{Fold\;Increase }=\frac{\mathrm{Bits\;per\;Synthesis\;Cycle}}{Bits\;per\;Synthesis\;Cycle\;of\;Standard\;Scheme}$$

### Ethics declaration

No animal or human subjects were involved in the study.

### Supplementary Information

Supplementary Information 1.

Supplementary Information 2.

Supplementary Video 1.

Supplementary Information 3.

Supplementary Information 4.

## Data Availability

The raw data is available in ENA (European Nucleotide Archive). The datasets generated and/or analyzed during the current study are available in the ENA (European Nucleotide Archive) repository, Accession Number—ERR12364864.

## Code Availability

Implementation of the algorithms and instructions on how to use them can be found in the GitHub repository in the following links: https://github.com/InbalPreuss/dna_storage_shortmer_simulation, https://github.com/InbalPreuss/dna_storage_experiment.

## References

[1] Church, G., Gao, Y. & Kosuri, S. Next-generation digital information storage in DNA. *Science* **337**, 1628 (2012). 22903519 10.1126/science.1226355

[2] Goldman, N. *et al.* Towards practical, high-capacity, low-maintenance information storage in synthesized DNA. *Nature* **494**, 77–80 (2013). 23354052 10.1038/nature11875 PMC3672958

[3] Anavy, L., Vaknin, I., Atar, O., Amit, R. & Yakhini, Z. Data storage in DNA with fewer synthesis cycles using composite DNA letters. *Nat. Biotechnol.* **37**, 1229–1236 (2019). 31501560 10.1038/s41587-019-0240-x

[4] Erlich, Y. & Zielinski, D. DNA Fountain enables a robust and efficient storage architecture. *Science* **355**, 950–954 (2017). 28254941 10.1126/science.aaj2038

[5] Gabrys, R., Kiah, H., & Milenkovic, O. Asymmetric lee distance codes for DNA-based storage. In *2015 IEEE International Symposium on Information Theory (ISIT)* (2015).

[6] NallappaBhavithran, G., & Selvakumar, R. Indel Error Correction Codes for DNA Digital Data Storage and Retrieval. ArXiv abs/2302.1467 (2023).

[7] Wang, C. *et al.* Mainstream encoding–decoding methods of DNA data. *CCF Trans. High Perform. Comput.* **4**, 23–22 (2022). 10.1007/s42514-022-00094-z

[8] Boruchvosky, A., Bar-Lev, D., & Yaakobi, E. DNA-Correcting Codes: End-to-end Correction in DNA Storage Systems. ArXiv, abs/2304.0391 (2023).

[9] Bornholt, J. *et al.* Toward a DNA-based archival storage system. *IEEE Micro* **37**, 98–104 (2017). 10.1109/MM.2017.70

[10] Yazdi, S., Yuan, Y., Ma, J., Zhao, H. & Milenkovic, O. A rewritable, random-access DNA-based storage system. *Sci. Rep.* **5**, 1–10 (2015). 10.1038/srep14138 PMC4585656 26382652

[11] Organick, L. *et al.* Random access in large-scale DNA data storage. *Biotechnol.* **36**, 242–248 (2018). 10.1038/nbt.4079 29457795

[12] Choi, Y. *et al.* High information capacity DNA-based data storage with augmented encoding characters using degenerate bases. *Sci. Rep.* **9**, 6582 (2019). 31036920 10.1038/s41598-019-43105-w PMC6488701

[13] Roquet, N., Bhatia, S., Flickinger, S., Mihm, S., Norsworthy, M., Leake, D., & Park, H. DNA-based data storage via combinatorial assembly. 20 April 2021 (online). 10.1101/2021.04.20.440194v1.

[14] Yan, Y., Pinnamaneni, N., Chalapati, S., Crosbie, C. & Appuswamy, R. Scaling logical density of DNA storage with enzymatically-ligated composite motifs. *Sci. Rep.* **13**, 15978 (2023). 37749195 10.1038/s41598-023-43172-0 PMC10519978

[15] LeProust, E. *et al.* Synthesis of high-quality libraries of long (105mer) oligonucleotides by a nover depurination controlled process. *Nucl. Acids Res.* **38**, 2522–2540 (2019). 10.1093/nar/gkq163 PMC2860131 20308161

[16] Barrett, M. *et al.* Comparative genomic hybridization using oligonucleotide microarrays and total genomic DNA. *Proc. Natl Acad. Sci. USA* **101**, 17765–17770 (2004). 15591353 10.1073/pnas.0407979101 PMC535426

[17] Eleuteri, A., Capaldi, D., Douglas, L. & Ravikumar, V. Oligodeoxyribonucleotide phosphorothioates: Substantial reduction of (N-1)-mer content through the use of trimeric phosphoramidite synthons. *Nucleosides Nucleotides* **3**, 475–483 (1999). 10.1080/15257779908043091

[18] Yagodkin, A. *et al.* Improved synthesis of trinucleotide phosphoramidites and generation of randomized oligonucleotide libraries. *Nucleosides Nucleotides Nucl. Acids* **26**(5), 473–497 (2007). 10.1080/15257770701426260 17578745

[19] Randolph, J., Yagodkin, A. & Mackie, H. Codon-based Mutagenesis. *Nucl. Acids Symp. Ser.* **52**, 479 (2008). 10.1093/nass/nrn243

[20] Ferrante, M., & Saltalamacchia, M. *The Coupon Collector’s Problem*, p 35 (2014).

[21] Press, W. *et al.* HEDGES error-correcting code for DNA storage corrects indels and allows sequence constraints. *Proc. Natl. Acad. Sci.* **117**(31), 18489–18496 (2020). 32675237 10.1073/pnas.2004821117 PMC7414044

[22] Haoling, Z., *et al*. SPIDER-WEB generates coding algorithms with superior error tolerance and real-time information retrieval capacity. arXiv preprint arXiv 2204.02855 (2022).

[23] Sabary, O., Orlev, Y., Shafir, R. & Anavy, L. SOLQC: Synthetic oligo library quality control tool. *Bioinformatics* **2**, 740 (2020). 10.1093/bioinformatics/btaa740 32840559

[24] Preuss, I., Galili, B., Yakhini, Z., & Anavy, Z. *Sequencing coverage analysis for combinatorial DNA-based storage systems*. *biorxiv* (2024).

[25] Ayoub, R. Euler and the zeta function. *Am. Math. Mon.* **81**, 1067–1086 (1974). 10.1080/00029890.1974.11993738

[26] Reed, I. & Solomon, G. Polynomial codes over certain finite fields. *J. Soc. Ind. Appl. Math.* **8**, 300–304 (1960). 10.1137/0108018

[27] Hipp, R. D. SQLite (2020) (Online). https://www.sqlite.org/index.html.

[28] Levenshtein, V. Binary codes capable of correcting spurious insertions and deletions of ones. *Problems Inf. Transm.* **1**, 8–17 (1965).

[29] Levenshtein, V. Binary codes capable of correcting deletion, insertions and reversals. *Soviet Physics Doklady* **10**(8), 707–710 (1966).

