# Supplementary material for: Efficient DNA-based data storage using shortmer combinatorial encoding
Source: Sci Rep. 2024 Apr 2;14:7731. doi: 10.1038/s41598-024-58386-z (PMC11369284; doi:10.1038/s41598-024-58386-z)
Supplement: Supplementary file 2 — Supplementary Information 2. [file 41598_2024_58386_MOESM2_ESM.pdf]

8 Supplementary

All files are available here.

8.1 Combinatorial shortmer synthesis (video)

Supplementary video uploaded separately, in:
Supplementary_animation_3-mers_16choose2.mp4

8.2 Bionomial shortmer alphabet example (table)

Supplementary file, uploaded separately in:
Supplementary_table_alphabet.xlsx

$\boldsymbol{N = |\Omega| = 16, K = 5}$. **The Hamming distance of this** Ω **is** $\boldsymbol{d = 2}$. $\boldsymbol{|\Sigma| = 4096 \leq 4368 = \binom{16}{5}}$

8.3 Example of k-mer sets

Supplementary Table S1 is an example of k-mer sets. To the left, are two sets of trimers that have a minimal Hamming distance of 2, with $|\Omega_1| = 16$ *and* $|\Omega_2| = 12$. To the right, is a set of 54 20-mers that have a minimal Hamming distance of 10.

**Trimers**		**20-mers**	
Ω_1	Ω_2	Ω_3	
AAT	ACG	GCGCCATTCTGATATTCACG	ACCTACCTTGTAATTGACGG
ACA	AAA	TAAGTTGTCGGAGCGACCTC	ACTGTCATGCATGCCTGAGA
ATG	AGC	CTAAGTGATCCGAGTGGCAT	CGTCAAGATAGCTAGTGACT
AGC	ATT	AAGGACTTGGCAGACCTCTG	CTGAGGAACGGTCGTTCATA
TAA	CAC	AATGGATTCCGTAACTATCG	AGGACTCACTCCAGGTATAC
TCT	CCA	GAATCGGCTATGCTGTCAGA	TTGCAAGCCTACCGTAGCGA
TTC	GAG	GCCGCTAGTACTAACCTGTG	GGTGTCCGGAATCGAATCCT
TGG	GCC	TTCGATGCGCACTGACATGA	CTTGACACGGTGGCATAACA
GAG	GGA	CGTGACGGTGCATTACTCCA	GTCTGGCCTAATCGTTAAGG
GCC	TAT	GTTACCAACACTGCTAGCGG	CCGCGCGAGCAATTACTTAT
GTT	TTA	TGCAGCCTACGCACTTAGTT	TCTCTGTGCAAGGCCATGAC
GGA		CGATACTCGTTGAACGATGC	CAACCTTAGGAGGTGCTCGC
CAC		TAAGCTCTATGGCCGCTGAG	ATTACAAGTCTGTGCCAGAG
CCG		TCTGAGTAGTGGCGACAACA	CATACCGTGAAGTCGGCGTA
CTA		AGCAGGACCTAACTGTGTAC	
CGT		GTACATCTCAGCCTGAAGTC	

Table S1: Example of k-mer sets, $\boldsymbol{\Omega}$**.** For $\boldsymbol{\Omega_1}$ and $\boldsymbol{\Omega_2}$, the minimum Hamming distance is 2. For $\boldsymbol{\Omega_3}$, the minimum hamming distance is 10.

8.4 Reconstruction of a binomial sequence

		Probability of unsuccessful reconstruction (δ)			
		10^{-2}	10^{-3}	10^{-4}	10^{-5}
Sequence length (l)	50	464	1445	4546	14351
	100	652	2039	6425	20291
	150	795	2495	7866	24848
	200	917	2879	9081	28691

Table S2: Sufficient number of reads to reconstruct a binomial sequence. Entries in the table represent $\boldsymbol{KHar(K) + cK}$ where $\boldsymbol{c}$ is derived based on the desired $\boldsymbol{\delta}$, as explained in Section **Error! Reference source not found.**.

8.5 Logical density and cost analysis per encoding

Logical density and cost analysis per encoding, uploaded separately: Logical_density_and_cost_analysis_per_encoding.xlsx

8.6 Proof-of-concept experimental design (Table)

Supplementary file uploaded separately, in: Supplementary_table_oligo_sequences.xlsx

8.7 Proof of concept smaller-scale experiment

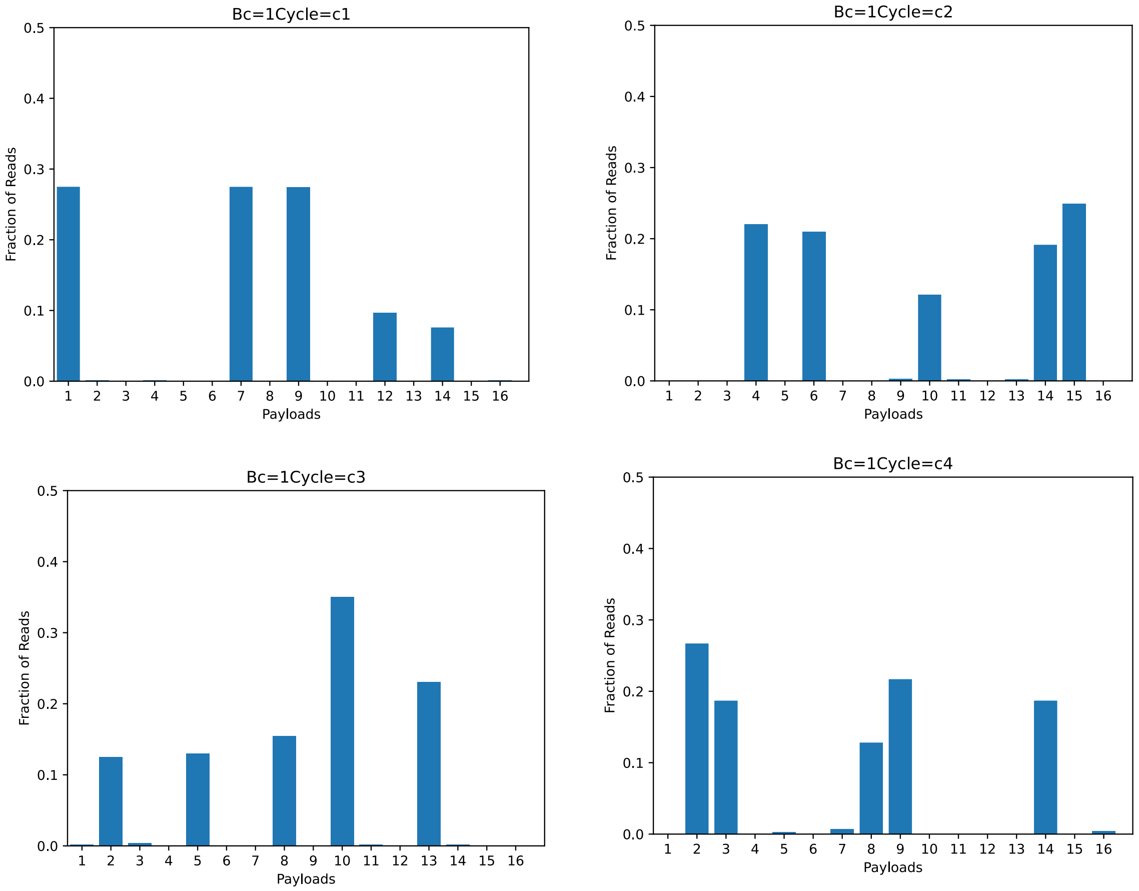

Fig. S1: Smaller-scale experiment, oligo 1. Oligo has four cycles, each with five inferred k-mers. Results showed the k-mers expected, according to our original design.

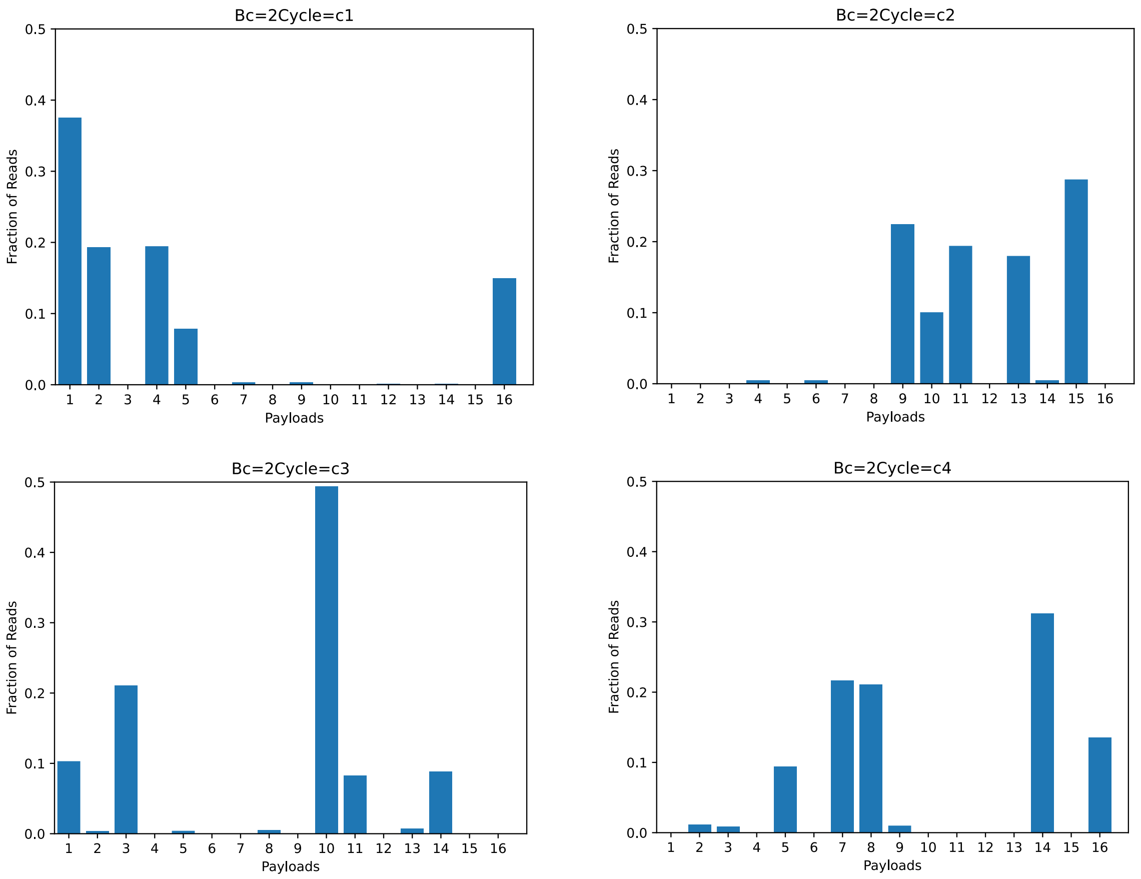

Fig. S2: Smaller-scale experiment, oligo 2. Oligo has four cycles, each with five k-mers. Results showed the k-mers expected, according to our original design.

For each of the two barcodes, we were able to identify and recover the barcode and their payloads. At each position/cycle in the sequence, the five-member k-mers were recovered, which are the five inferred k-mers (See Supplementary Fig. S1 and Supplementary Fig. S2).

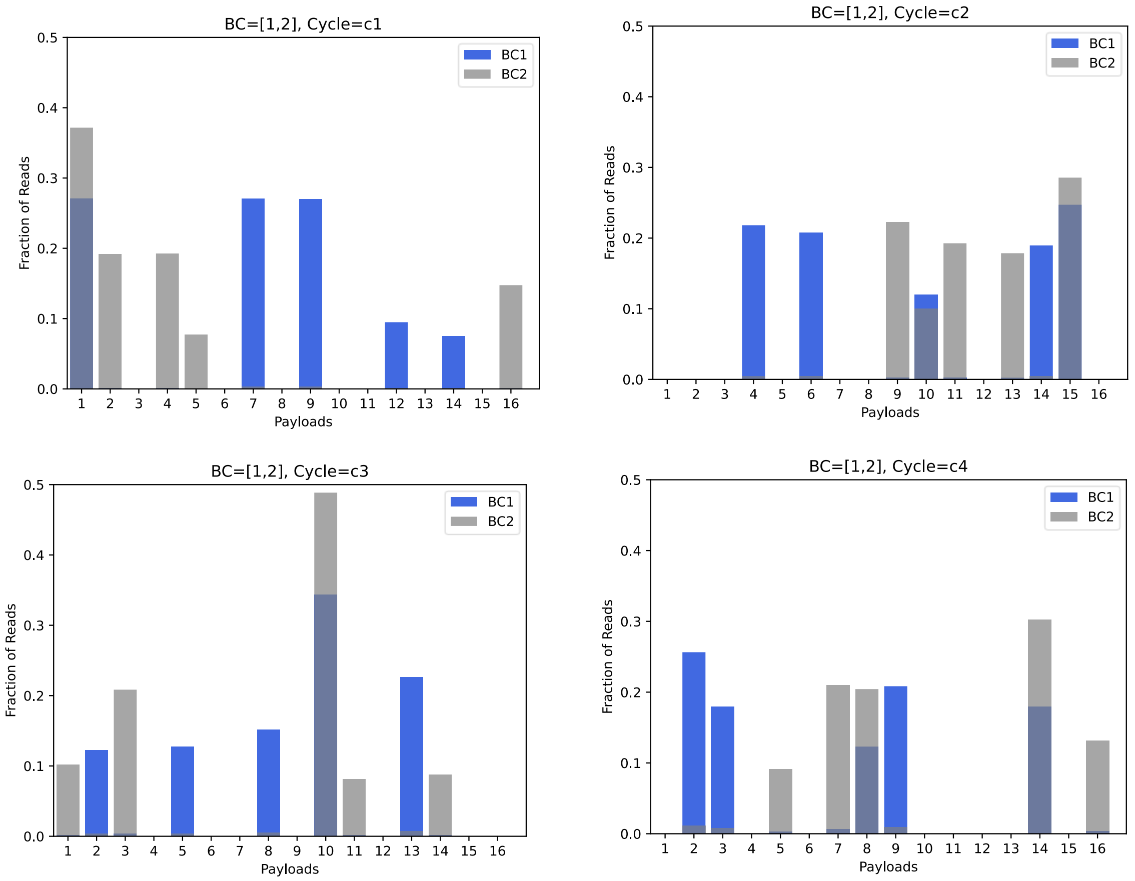

Fig. S3: Superimposing oligo 2 (grey) over oligo 1 (blue). Leakage resulted in more than five k-mers expected, according to our design, where k-mers continued to assemble in each cycle due to the active enzyme, yet not necessarily on their designated oligo. The same applies to the second sequence that was assembled. Note that there is a partial overlap between the member k-mers of the two sequences, for example, Payloads #10 and #15 in cycle C2.
